# Melatonin downregulates TRPC6, impairing store-operated calcium entry in triple-negative breast cancer cells

**DOI:** 10.1074/jbc.RA120.015769

**Published:** 2021-01-08

**Authors:** Isaac Jardin, Raquel Diez-Bello, Debora Falcon, Sandra Alvarado, Sergio Regodon, Gines M. Salido, Tarik Smani, Juan A. Rosado

**Affiliations:** 1 Department of Physiology (Cellular Physiology Research Group), Institute of Molecular Pathology Biomarkers (IMPB), University of Extremadura, Caceres, Spain; 2 Cardiovascular Physiopathology Group, Institute of Biomedicine of Sevilla, Sevilla, Spain; 3 Department of Animal Medicine, University of Extremadura, Caceres, Spain; 4 Department of Medical Physiology and Biophysic, Institute of Biomedicine of Sevilla, Sevilla, Spain

**Keywords:** melatonin, TRPC6, triple-negative breast cancer cells, calcium entry, SOCE, BrdU, bromodeoxyuridine, BSA, bovine serum albumin, ER, endoplasmic reticulum, HBS, HEPES-buffered saline, HER2, human epidermal growth factor receptor 2, OAG, 1-oleoyl-2-acetyl-sn-glycerol, OASF, Orai1-activating small fragment, PI, propidium iodide, SOCE, store-operated Ca^2+^ entry, TG, Thapsigargin, TNBC, triple-negative breast cancer

## Abstract

Melatonin has been reported to induce effective reduction in growth and development in a variety of tumors, including breast cancer. In triple-negative breast cancer (TNBC) cells, melatonin attenuates a variety of cancer features, such as tumor growth and apoptosis resistance, through a number of still poorly characterized mechanisms. One biological process that is important for TNBC cells is store-operated Ca^2+^ entry (SOCE), which is modulated by TRPC6 expression and function. We wondered whether melatonin might intersect with this pathway as part of its anticancer activity. We show that melatonin, in the nanomolar range, significantly attenuates TNBC MDA-MB-231 cell viability, proliferation, and migration in a time- and concentration-dependent manner, without having any effect on nontumoral breast epithelial MCF10A cells. Pretreatment with different concentrations of melatonin significantly reduced SOCE in MDA-MB-231 cells without altering Ca^2+^ release from the intracellular stores. By contrast, SOCE in MCF10A cells was unaffected by melatonin. In the TNBC MDA-MB-468 cell line, melatonin not only attenuated viability, migration, and SOCE, but also reduced TRPC6 expression in a time- and concentration-dependent manner, without altering expression or function of the Ca^2+^ channel Orai1. The expression of exogenous TRPC6 overcame the effect of melatonin on SOCE and cell proliferation, and silencing or inhibition of TRPC6 impaired the inhibitory effect of melatonin on SOCE. These findings indicate that TRPC6 downregulation might be involved in melatonin's inhibitory effects on Ca^2+^ influx and the maintenance of cancer hallmarks and point toward a novel antitumoral mechanism of melatonin in TNBC cells.

Breast cancer is one of the most common tumors and the major cause of female cancer deaths in the Western world (1). Among the breast cancer subtypes, triple-negative breast cancer (TNBC) is characterized by the absence of estrogen and progesterone receptors and lack of excess of human epidermal growth factor receptor 2 (HER2) expression. TNBC accounts for about 15% of all breast cancer cases (2), and TNBC cells exhibit low levels of differentiation as well as rapid and aggressive proliferation (3). A number of cancer features in TNBC cells have been reported to depend on Ca^2+^ influx across the plasma membrane. A major pathway for Ca^2+^ influx in TNBC cells is store-operated Ca^2+^ entry (SOCE), a mechanism regulated by the filling state of the agonist-sensitive intracellular Ca^2+^ stores. SOCE in TNBC cells has been demonstrated to occur with the participation of the endoplasmic reticulum (ER) Ca^2+^ sensor STIM1 and the Ca^2+^ permeable channel Orai1 (4). The latter is a well-known regulator of proliferation and migration of TNBC cells (5, 6). We have recently reported that the cation permeable channel TRPC6 interacts with Orai1 in the triple-negative MDA-MB-231 cell line, where it is required for the expression of Orai1 in the plasma membrane and, thus, SOCE (7). TRPC6 has been found to be highly expressed in human breast ductal adenocarcinoma compared with the adjacent nontumor tissue (8, 9), as well as in a number of breast cancer cell lines (9, 10), including the TNBC cell line MDA-MB-231 (7, 9). The high TRPC6 expression in TNBC cells suggests that it might be relevant for cell function, and thus, TRPC6 might be a target for antitumoral therapeutic strategies. Concerning the functional role of TRPC6 in TNBC cells, it has been reported that functional TRPC6 is required for cell proliferation and both 2D and 3D migration (7, 9). Consistent with this, treatment of MDA-MB-231 cells with the olive-oil-derived phenolic compound oleocanthal results in attenuation of TRPC6 expression and, consequently, reduced cell proliferation, migration, and viability, an effect that was absent in the nontumoral breast epithelial MCF10A cell line (11).

Melatonin (N-acetyl-5-methoxy tryptamine) has been reported to exhibit effective reduction in growth and development in a variety of tumors (12, 13, 14), including breast cancer (15, 16). The antitumoral actions of melatonin on breast cancer cells have been widely studied in cells of the luminal (estrogen receptor expressing) subtype (17, 18). In TNBC, melatonin attenuates tumor growth (19) as well as the expression of NF-κB (20) in MDA-MB-231 cells in a xenograft tumor model. Furthermore, *in vitro* studies have reported that treatment with melatonin, in the millimolar range, decreases viability in MDA-MB-231 cells by inducing apoptosis under acute acidosis conditions (21). Melatonin has been reported to attenuate MDA-MB-231 cell invasion and migration in the nanomolar range, a mechanism that has been shown to be mediated by inhibition of the DJ-1/KLF17/ID-1 signaling pathway (22), as well as by the expression of kisspeptin (KiSS1), a well-known suppressor of metastasis (23).

A role for melatonin in the modulation of Ca^2+^ influx has been reported. Melatonin reduces dopamine release in rat hypothalamus by attenuation of Ca^2+^ entry (24) and exerts neuroprotective effects on rat hippocampus by attenuating Ca^2+^ entry *via* TRPM2 (25). Furthermore, administration of melatonin restores SOCE in aged mice pancreatic acinar cells (26) and enhances thrombin-evoked Ca^2+^ entry and aggregation in sheep platelets (27). In the present study, we have investigated the possible modulation of TRPC6 function by melatonin and the role of SOCE in the antitumoral effects of melatonin in TNBC cells.

## Results

### Effect of melatonin in MDA-MB-231 and MCF10A cell viability and migration

**Figure 1:**
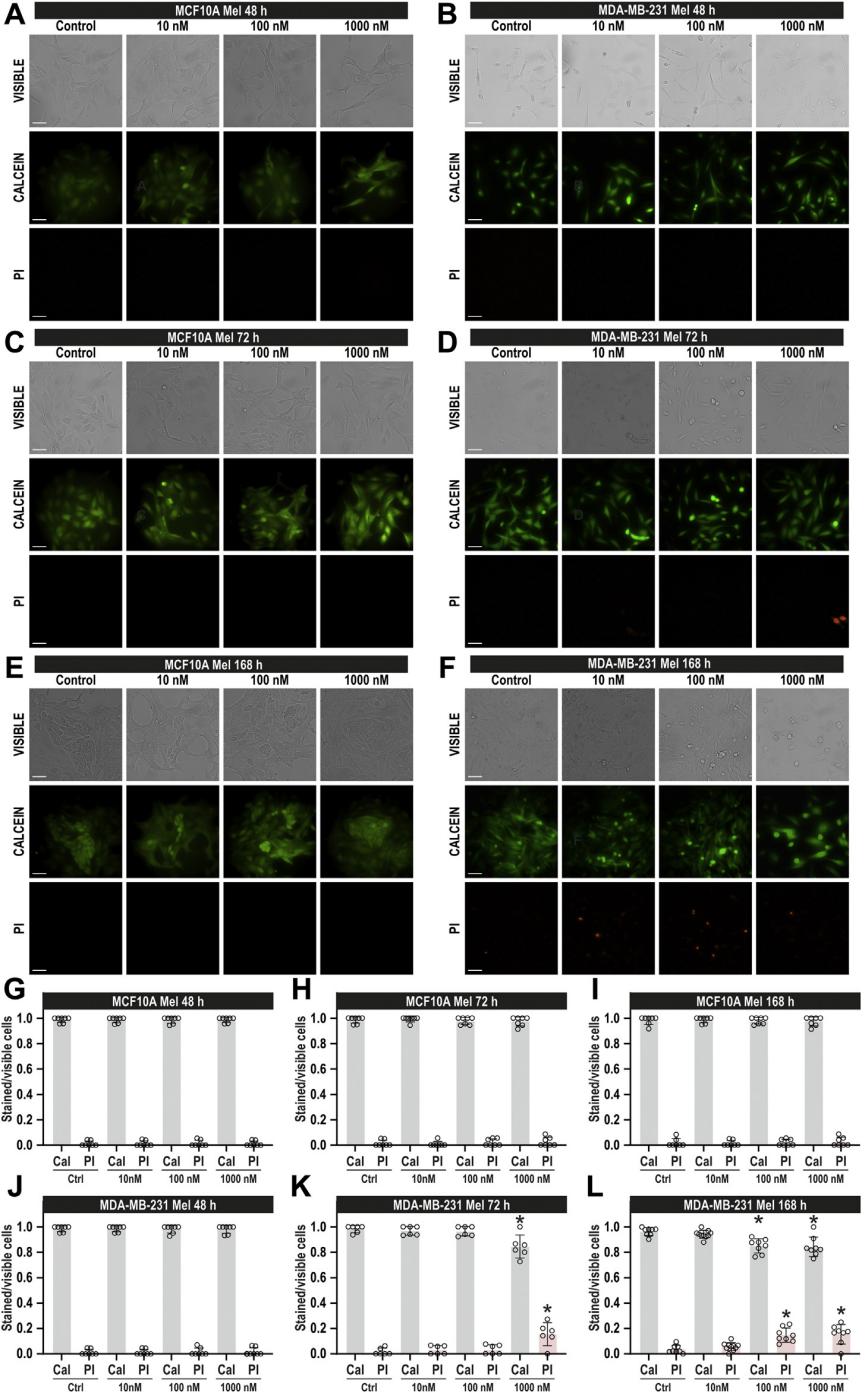
**Melatonin attenuates MDA-MB-231 cell viability.** MCF10A (*A*, *C* and *E*) and MDA-MB-231 cells (*B*, *D*, and *F*) were treated with melatonin (10–1000 nM) or the vehicle, and 48, 72, or 168 h later, cells were loaded with calcein and propidium iodide (PI). Cell staining was visualized using an inverted microscope as described in Experimental procedures. *G*–*L*, scatter plots represent calcein (Cal) and propidium iodide (PI) staining under the different conditions expressed as the ratio between stained *versus* visible cells and are expressed as mean ± SD. Images shown are representative of 6–8 independent experiments. Analysis of statistical significance was performed using two-way ANOVA (F values for calcein and PI data were 1.34, 2.01, and 0.61 and *p* values were 0.26, 0.14, and 0.71 for concentration, time, and the interaction, respectively, for *G*, *H*, and *I*. F values for calcein and PI data were 106.5, 179.8, and 38.53, and *p* values were <0.0001 for concentration, time, and the interaction respectively for *J*, *K*, and *L*) with post-hoc Tukey's multiple comparison test (*p* <0.0001 for *J*–*L*). ∗*p* < 0.05 as compared with their respective controls (Dunnett's test).

Melatonin exerts antitumoral effects in a number of cancer cells and tumoral tissues, including breast cancer (20, 22). Hence, we have explored the effect on cell viability of the treatment of TNBC MDA-MB-231 cells and nontumoral breast epithelial MCF10A cells with melatonin by using the cell-permeant dye calcein and propidium iodide. MDA-MB-231 and MCF10A cells were treated with increasing concentrations of melatonin (10–1000 nM) or the vehicle, as control, and 48, 72, and 168 h (1 week) later, calcein and propidium iodide fluorescences were assessed. As shown in Figure 1, *A*, *C*, and *E*, our results indicate that almost 100% of the MCF10A cells show that calcein fluorescence and propidium iodide staining were unaffected by treatment with melatonin at any of the concentrations tested, at least for 1 week (n = 7). Treatment of MDA-MB-231 cells with melatonin only significantly attenuates calcein staining after pretreatment with 1000 nM for 72 h (*p* = 0.0008; one-way ANOVA combined with Dunnett's test) and 168 h (*p* = 0.0002; Dunnett's test) or after treatment with 100 nM for 168 h (*p*= 0.0004; Dunnett's test; see Fig. 1). Consistently, pretreatment with 100 nM melatonin for 168 h (*p* = 0.0004; Dunnett's test) or with 1000 nM melatonin for 72 h (*p* = 0.0008) and 168 h (*p* = 0.0002) significantly enhanced propidium iodide staining (Fig. 1; n = 6–8). These findings indicate that melatonin reduces MDA-MB-231 cell viability in a time- and concentration-dependent manner without affecting the viability of MCF10A cells.

**Figure 2:**
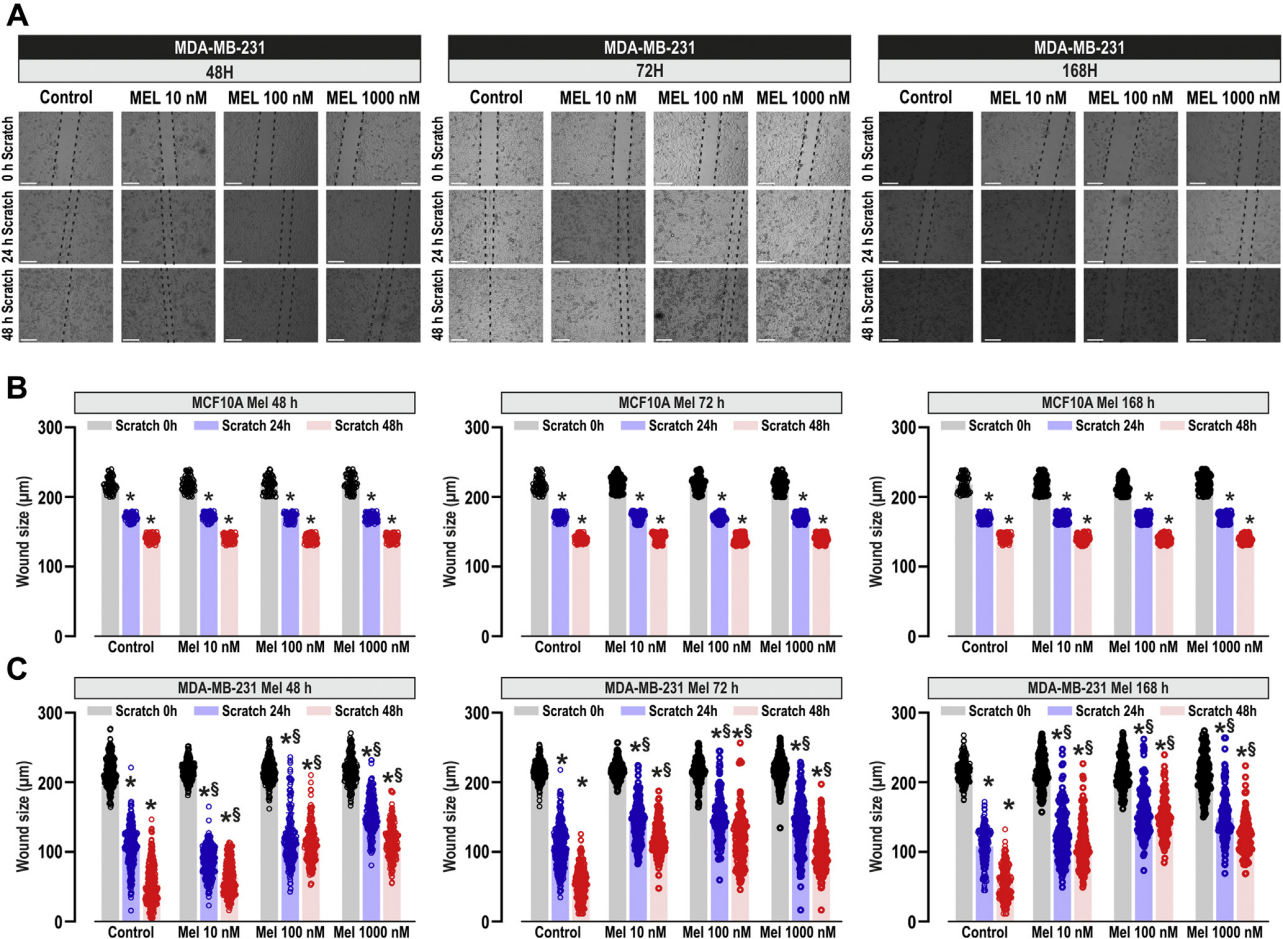
**Melatonin attenuates MDA-MB-231 cell migration.** MCF10A and MDA-MB-231 cells were treated for 48, 72, or 168 h with melatonin (10–1000 nM) or the vehicle (control) and were subjected to wound healing assay as described in Experimental procedures. *A*, representative images were acquired at 0, 24, and 48 h from the beginning of the assay. Bars represent 200 μm. The *dotted lines* define the areas lacking cells. *B* and *C*, quantification of the wound size, in micrometers, at the different conditions, expressed as the mean ± SD of four independent experiments. Analysis of statistical significance was performed using two-way ANOVA (for *B*: 0 h, F values were 0.21, 0.02, and 0.58 and *p* values were 0.88, 0.97, and 0.73 for concentration, time, and the interaction respectively; for *B*: 24 h, F values were 1.11, 0.60, and 1.14 and *p* values were 0.34, 0.54, and 0.33; for *B*: 48 h, F values were 1.72, 0.86, and 0.62 and *p* values were 0.16, 0.42, and 0.71; for *C*: 0 h, F values were 0.35, 0.31, and 0.17 and *p* values were 0.78, 0.72, and 0.98; for *C*: 24 h, F values were 1570, 616.6, and 447.6 and *p* values were <0.0001 in all cases; for *C*: 48 h, F values were 6019, 958.4, and 608.1 and *p* values were <0.0001 in all cases) with post-hoc Tukey test (∗*p* < 0.0001 compared with the scratch time = 0 h. ^$^*p* < 0.01 compared with the corresponding time in vehicle-treated cells).

We have further explored the effect of melatonin on MCF10A and MDA-MB-231 cells migration using the well-established wound healing assay (28). Cells were seeded, manually scratched, and cultured in culture medium, supplemented with 1% serum to prevent further cell growth, and cell migration was determined as described in Experimental procedures. MCF10A and MDA-MB-231 cells were treated with increasing concentrations of melatonin (10–1000 nM), or the vehicle (Control), for 48, 72, and 168 h, and then scratched. The wound size was determined 0, 24, and 48 h after scratching, as indicated (Fig. 2*A*). In the absence of melatonin, all the cells significantly reduced the wound size during the first 48 h (Fig. 2; *p* < 0.05; n = 4). Addition of melatonin to MCF10A cells did not significantly attenuate cell migration at the times and concentrations investigated (Fig. 2*B*; n = 4). By contrast, treatment of MDA-MB-231 cells with melatonin significantly attenuated cell migration in a concentration-dependent manner (Fig. 2, *A* and *C*; *p* < 0.05; n = 4). The effect observed after 168 h of treatment at any concentration of melatonin or after 72 h with 1000 nM melatonin might be attributed to a reduction in cell viability (as shown in Fig. 1), but the inhibitory effects of melatonin at 10 or 100 nM for 48 or 72 h pretreatment (Fig. 2*C*, left and middle panel) cannot be attributed to changes in cell viability, thus suggesting that treatment with melatonin impairs TNBC cell migration without affecting the ability of MCF10A cells to migrate.

### Melatonin attenuates store-operated Ca^2+^ entry in MDA-MB-231 cells

**Figure 3:**
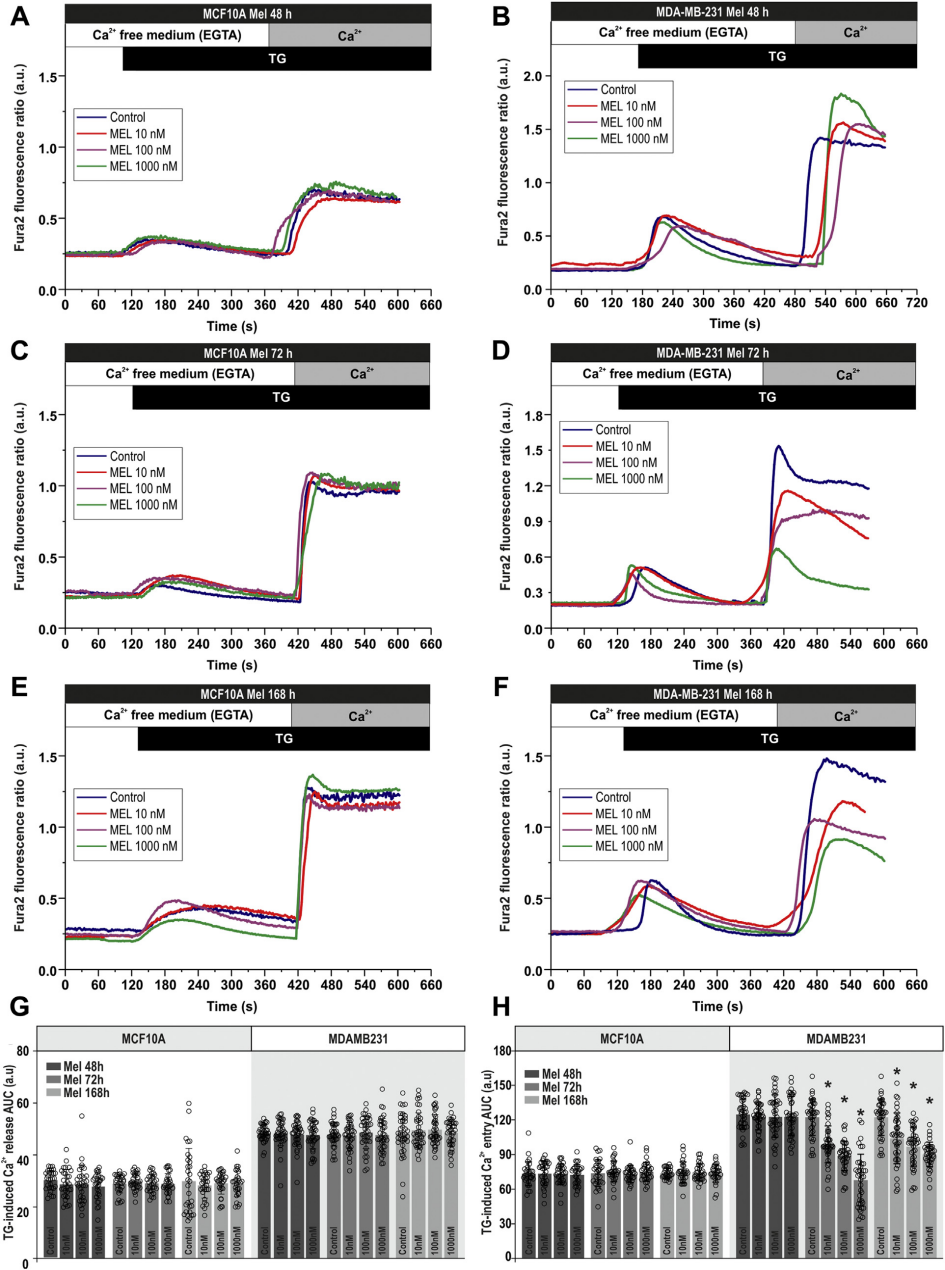
**Melatonin attenuates store-operated Ca**^**2+**^**entry but not Ca**^**2+**^**release in MDA-MB-231 breast cancer cells.***A*–*F*, MCF10A (*A*, *C*, and *E*) and MDA-MB-231 (*B*, *D*, and *F*) cells were treated for 48, 72, or 168 h with melatonin (10–1000 nM) or the vehicle (control). Fura-2-loaded cells were perfused with a Ca^2+^-free medium (100 μM EGTA added) and then stimulated with TG (1 μM) followed by reintroduction of external Ca^2+^ (final concentration 1 mM) to initiate Ca^2+^ entry. *G* and *H*, quantification of TG-evoked Ca^2+^ release (*G*) or entry (*H*) determined as described in Experimental procedures. Scatter plots are represented as mean ± SD. *Dots* represent single experiments including 20–30 cells. Analysis of statistical significance was performed using two-way ANOVA (F values were 0.97, 0.20, and 0.85 and *p* values were 0.40, 0.81, and 0.52 for concentration, time, and the interaction respectively for Ca^2+^ release in MCF10A; F values were 2.73, 0.40, and 5.54 and *p* values were 0.64, 0.91, and 0.79 for Ca^2+^ release in MDAMB231; F values were 1989, 1777, and 1145 and *p* values were 0.11, 0.17, and 0.33 for Ca^2+^ entry in MCF10A; F values were 459.4, 891.8, and 125.8 *p* values were <0.0001 in all cases for Ca^2+^ entry in MDAMB231) with post-hoc Tukey test (∗*p* < 0.0001 compared with the response observed in vehicle-treated cells).

Since Ca^2+^ entry has been reported to play a relevant role supporting different cancer hallmarks in TNBC cells (4, 7), we have assessed the ability of melatonin to impair SOCE in these cells. MCF10A and MDA-MB-231 cells were pretreated for 48, 72, and 168 h with increasing concentrations of melatonin (10–1000 nM), and SOCE was evoked by Ca^2+^ store depletion using the SERCA inhibitor TG. As depicted in Figure 3, cell treatment with TG (1 μM) resulted in a transient increase in cytosolic free-Ca^2+^ concentration due to Ca^2+^ release from the intracellular Ca^2+^ stores. Subsequent addition of CaCl_2_ (1 mM) to the extracellular medium resulted in a further increase in cytosolic free-Ca^2+^ concentration, indicative of SOCE. In MCF10A cells, treatment with melatonin did not significantly alter Ca^2+^ release or entry induced by TG (Fig. 3, *A*, *C*, *E*, and *G*; n = 50 cells/day/5–7 days). Interestingly, pretreatment of MDA-MB-231 cells with melatonin, exclusively for 72 or 168 h, significantly attenuated TG-induced Ca^2+^ influx in a concentration-dependent manner, without having any significant effect on Ca^2+^ release from the intracellular stores (Fig. 3, *B*, *D*, *F*, and *H*; n = 50 cells/day/5–7 days). Altogether, these findings indicate that melatonin specifically reduces the activation of SOCE in MDA-MB-231 breast cancer cells while it does not have detectable effect in nontumoral MCF10A cells.

### Melatonin attenuates TRPC6 expression in MDA-MB-231

**Figure 4:**
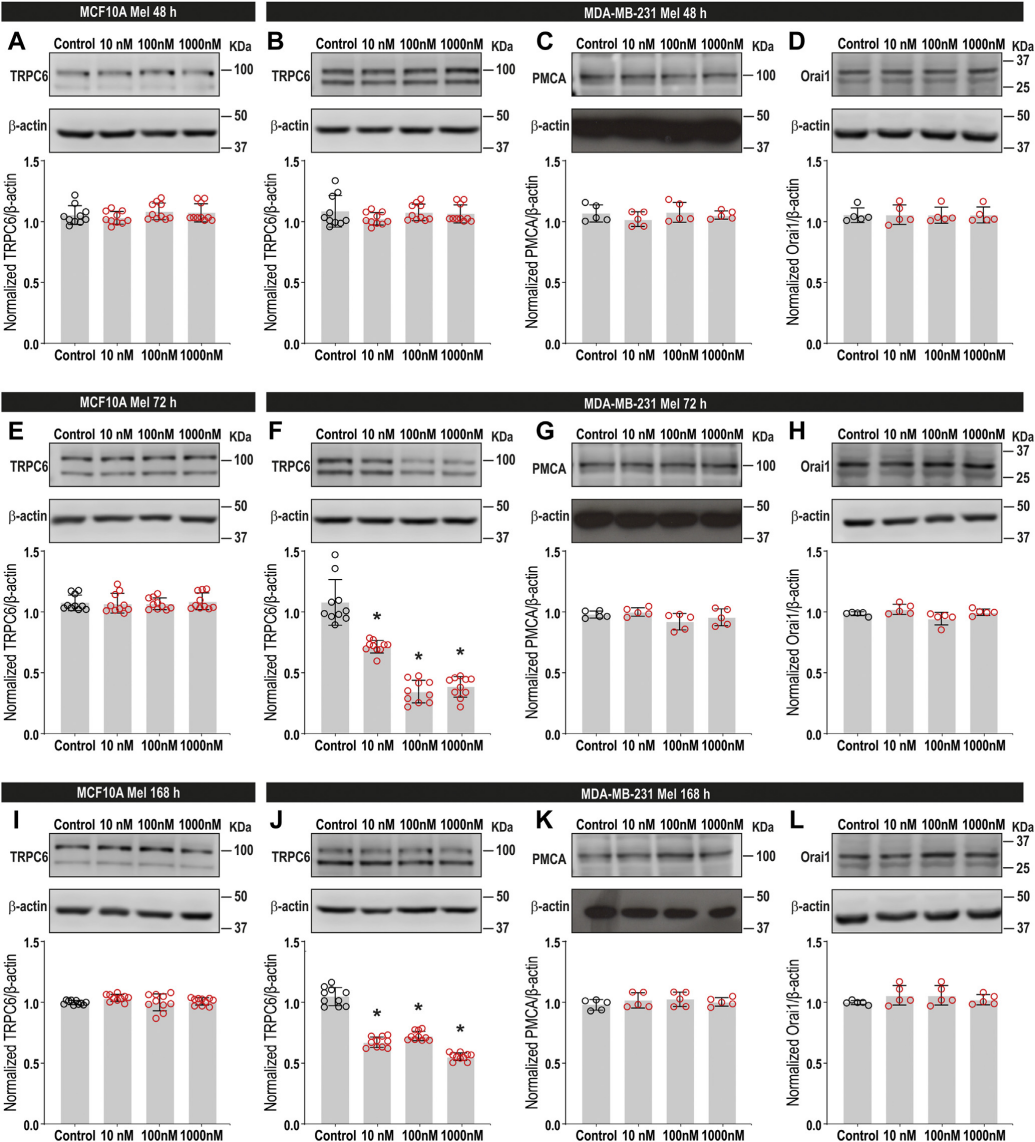
**Melatonin downregulates TRPC6 expression in MDA-MB-231 cells.** MCF10A (*A*, *E* and *I*) and MDA-MB-231 cells (*B*–*D*, *F*–*G* and *J*–*L*) were treated for 48, 72, and 168 h with melatonin (10–1000 nM) or the vehicle (control) and lysed. Whole-cell lysates were subjected to 10% SDS-PAGE and western blotting with the anti-TRPC6 (*A*, *B*, *E*, *F*, *I*, and *J*), anti-PMCA (*C*, *G*, and *K*), anti-Orai1 (*D*, *H* and *L*), or anti-β-actin antibodies, as described in Experimental procedures. Blots are representative of five to ten separate experiments. Scatter plots represent TRPC6, Orai1, or PMCA expression. Analysis of statistical significance was performed using one-way ANOVA (0.001 < F < 2.04 and 0.14 < *p* < 0.99 for all panels, except (*F*) (F = 86.34; *p* < 0.0001) and (*J*) (F = 182.2; *p* < 0.0001)) with post-hoc Dunnett's test for (*F* and *J*) (∗*p* < 0.0001 compared with control).

TRPC6 expression has been found to be enhanced in breast tumoral samples from patients as well as in both luminal breast cancer cell lines and TNBC cell lines (7, 9). In MDA-MB-231 cells, SOCE is highly dependent on TRPC6 expression (7). Hence, in order to ascertain the molecular basis of the effect of melatonin on SOCE in MDA-MB-231 cells, we have assessed its effect on TRPC6 channel expression. MCF10A and MDA-MB-231 cells were treated with increasing concentrations of melatonin (10–1000 nM) for 48, 72, or 168 h or the vehicle, as control, and then subjected to western blotting with specific anti-TRPC6 antibody. Interestingly, treatment of MDA-MB-231 cells with melatonin for 72 or 168 h significantly attenuated TRPC6 channel expression in a concentration-dependent manner as compared with vehicle-treated cells where the expression of TRPC6 was unaffected during the time investigated (Fig. 4, *F* and *J*; *p* < 0.0001; n = 8–10). No significant effect on TRPC6 expression was detected upon treatment with melatonin for 48 h (Fig. 4*B*; n = 8–10). By contrast, treatment of MCF10A cells with melatonin did not significantly alter TRPC6 expression at any concentration or pretreatment time investigated (Fig. 4, *A*, *E*, and *I*; n = 10). In order to assess whether melatonin has a nonspecific effect on plasma membrane proteins, we tested its effect on the PMCA expression in MDA-MB-231 cells. As shown in Figure 4, *C*, *G*, and K, melatonin was unable to alter the PMCA expression at any of the experimental procedures tested (n = 5). Due to the relevance of Orai1 in SOCE in MDA-MB-231 cells (4), we have further explored whether melatonin alters its expression at the time and concentrations tested. As depicted in Figure 4, *D*, *H*, and L, pretreatment with melatonin (10–1000 nM) for 48, 72, and 168 h was without effect on the expression of Orai1 (n = 5). These findings indicate that melatonin downregulates TRPC6 expression in MDA-MB-231 breast cancer cells, without having any effect on Orai1 expression, which might be responsible for the inhibition of cell viability and migration.

### Overexpression of TRPC6 attenuates the inhibitory effect of melatonin on SOCE in MDA-MB-231 cells

**Figure 5:**
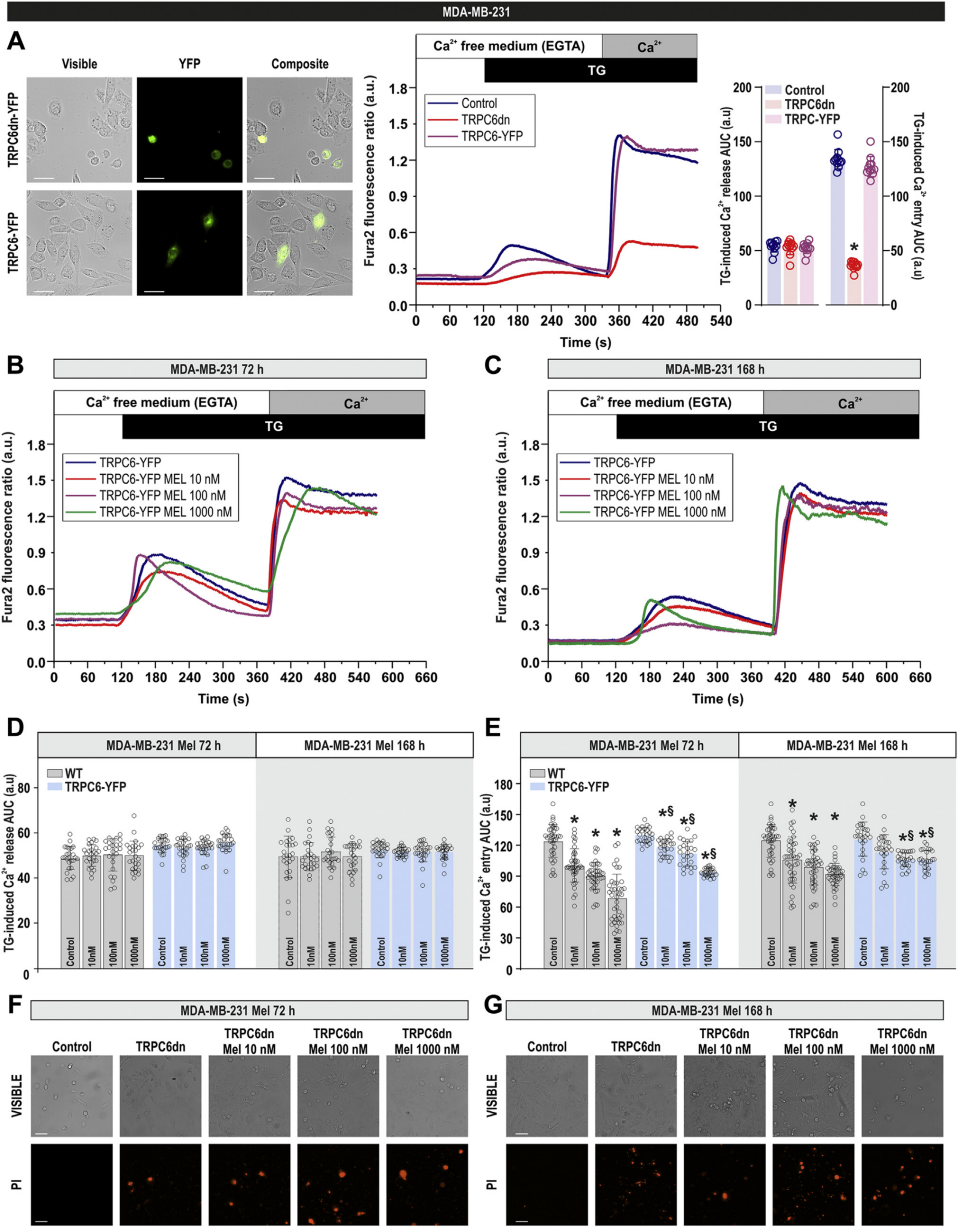
**Overexpression of TRPC6 attenuates melatonin-evoked inhibition of store-operated Ca**^**2+**^**entry in MDA-MB-231 cells.***A*, *left panel*, MDA-MB-231 cells were transfected with TRPC6dn-YFP or TRPC6-YFP expression plasmids. Forty-eight hours later, TRPC6 expression was determined by fluorescence microscopy. *A*, *right panel*, MDA-MB-231 cells were transfected with TRPC6dn-YFP or TRPC6-YFP expression plasmids or empty vector (control). Forty-eight hours later cells were loaded with fura-2. Fura-2-loaded cells were perfused with a Ca^2+^-free medium (100 μM EGTA added) and then stimulated with TG (1 μM) followed by reintroduction of external Ca^2+^ (final concentration 1 mM) to initiate Ca^2+^ entry. Scatter plots represent the quantification of TG-evoked Ca^2+^ release and entry determined as described in Experimental procedures. Data are presented as mean ± SD. *Dots* represent a single experiment including 20–30 cells. Analysis of statistical significance was performed using one-way ANOVA (F = 1.82; *p* = 0.19 for Ca^2+^ release and F = 546.5 and *p* < 0.0001 for Ca^2+^ entry, with post-hoc Dunnett's test; ∗*p* < 0.0001 compared with control). *B*–*E*, MDA-MB-231 cells were treated with melatonin (10–1000 nM) or the vehicle for 72 and 168 h. Forty-eight hours before the end of the treatment period, cells were transfected with TRPC6-YFP expression plasmid. Fura-2-loaded cells were perfused with a Ca^2+^-free medium (100 μM EGTA added) and then stimulated with TG (1 μM) followed by reintroduction of external Ca^2+^ (final concentration 1 mM) to initiate Ca^2+^ entry. *D* and *E*, quantification of TG-evoked Ca^2+^ release and entry. Scatter plots are represented as mean ± SD. *Dots* represent single experiments including 20–30 cells. Data obtained from cells transfected with TRPC6-YFP are compared with those obtained in control cells (*gray bars*; presented in Fig. 3) performed in parallel. Analysis of statistical significance was performed using two-way ANOVA (F values were 0.28, 0.59, and 0.32 and *p* values were 0.83, 0.61, and 0.96 for concentration, time, and the interaction respectively for Ca^2+^ release; F values were 769.6, 2479, and 80.87 and *p* values were <0.0001 in all cases for Ca^2+^ entry) with post-hoc Tukey test (∗*p* < 0.0001 compared with the response observed in vehicle-treated cells). *F*–*G*, MDA-MB-231 cells were treated with melatonin (10–1000 nM) or the vehicle for 72 and 168 h. Forty-eight hours before the end of the treatment period, cells were transfected with TRPC6dn-YFP expression plasmid or empty vector. Cells were loaded with propidium iodide, and cell staining was visualized using an inverted microscope as described in Experimental procedures. Images shown are representative of three independent experiments.

We have recently reported that TRPC6 knockdown significantly attenuates Ca^2+^ influx, as well as cell proliferation and migration in MDA-MB-231 cells (7). Hence, we have further investigated whether TRPC6 downregulation by melatonin underlies the effect of the hormone in SOCE in these cells by overexpressing TRPC6 or a pore-dead dominant-negative TRPC6 mutant (TRPC6dn) (7). As shown in Figure 5*A*, overexpression of TRPC6 has no additional effects on SOCE in MDA-MB-231 cells; by contrast, expression of TRPC6dn significantly inhibited SOCE. Interestingly, as depicted in Figure 5, *B* and *C*, in cells overexpressing TRPC6, the ability of melatonin to inhibit SOCE was significantly attenuated as compared with control cells (Fig. 3; for comparison see scatter plots in Fig. 5, *D* and *E*). These findings suggest that TRPC6 downregulation is at least partially involved in the inhibitory effect of melatonin on SOCE in MDA-MB-231 cells. Overexpression of TRPC6dn and treatment with melatonin resulted in a significant decrease in cell viability, which prevented SOCE determination. The percentage of cells stained with propidium iodide after overexpression of TRPC6dn, in the absence and presence of 10, 100, and 1000 nM melatonin, was 40 ± 3, 52 ± 4, 57 ± 2, and 61 ± 4% after treatment for 72 h with melatonin and 45 ± 4, 42 ± 1, 53 ± 3, and 65 ± 1% after treatment for 168 h with melatonin, respectively (Fig. 5, *F* and *G*; n = 3; analysis of statistical significance was performed using one-way ANOVA [F = 169.1 and 263.6 for Fig. 5*F* and Fig. 5*G*, respectively, *p* < 0.0001] combined with post-hoc Tukey test [*p* < 0.0001 as compared with control]).

### Overexpression of TRPC6 attenuates the inhibitory effect of melatonin on MDA-MB-231 cell proliferation

**Figure 6:**
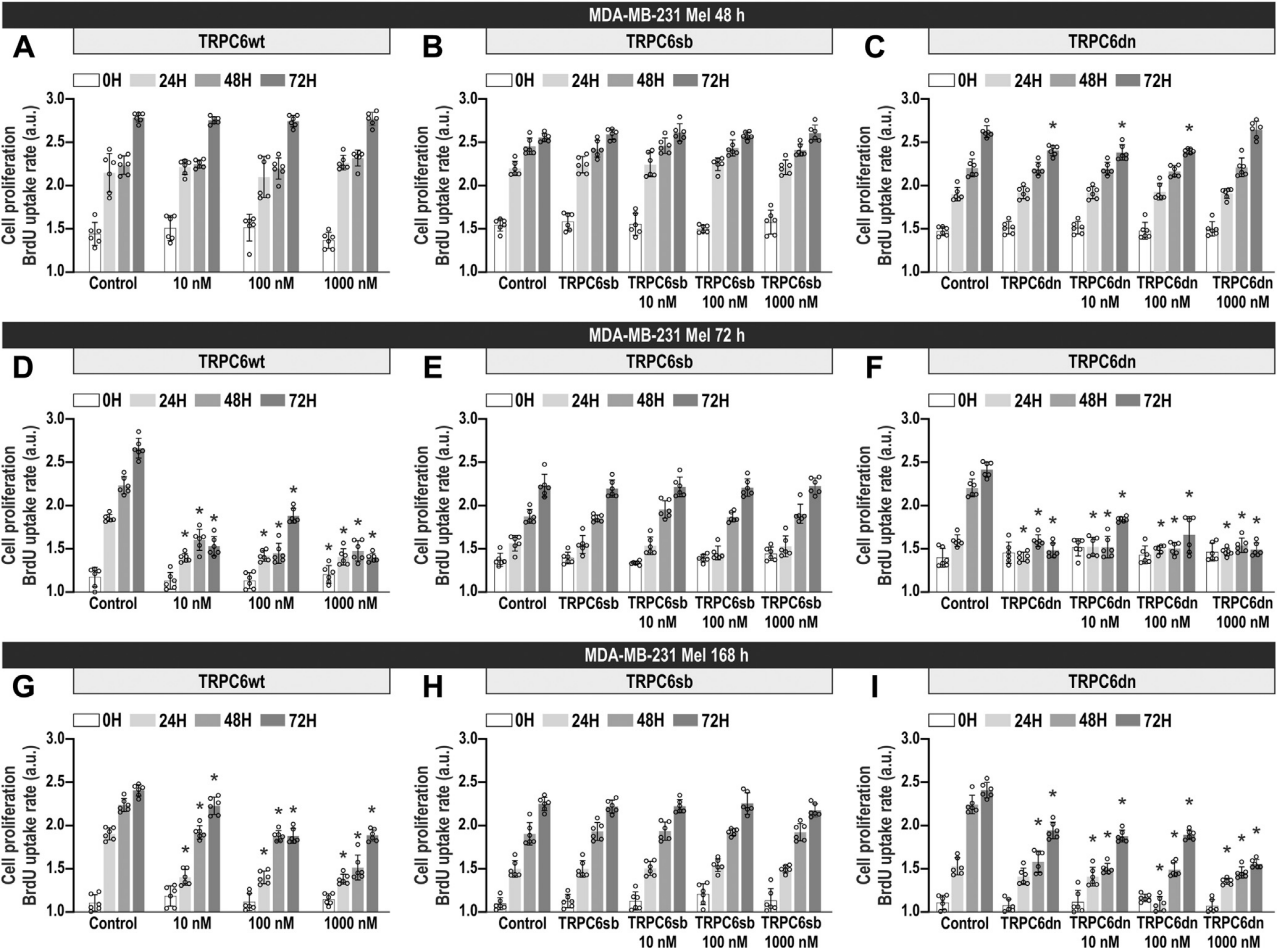
**Expression of exogenous TRPC6 reverses the antiproliferative effect of melatonin in MDA-MB-231 cells.** MDA-MB-231 cells were treated for 48, 72, and 168 h with melatonin (10–1000 nM) or the vehicle (control). Forty-eight hours before the end of the treatment period, cells were transfected with TRPC6-YFP (*B*, *E*, and *H*) or TRPC6dn-YFP (*C*, *F*, and *I*) expression plasmid or empty vector (*A*, *D* and *G*). Cell proliferation was assessed at time = 0, 24, 48, and 72 h using the BrdU cell proliferation assay kit, as described in Experimental procedures. Scatter plots represent cell proliferation 0, 24, 48, and 72 h after the onset of the experiment, presented as BrdU uptake rate (n = 6). Analysis of statistical significance was performed using two-way ANOVA (for *A*, *D*, *G*: 0 h, F values were 0.14, 0.14, and 1.35 and *p* values were 0.93, 0.86, and 0.24 for concentration, time, and the interaction respectively; for *A*, *D*, *G*: 24 h, F values were 593.8, 1570, and 199.6 and *p* values were <0.0001 in all cases; for *A*, *D*, *G*: 48 h, F values were 512.2, 1309, and 201.4 and *p* values were <0.0001 in all cases; for *A*, *D*, *G*: 72 h, F values were 1332, 5690, and 619.2 and *p* values were <0.0001 in all cases; for *B*, *E*, *H*: 0 h, F values were 0.03, 0.0.05, and 0.03 and *p* values were 0.99, 0.94, and 0.99; for *B*, *E*, *H*: 24 h, F values were 0.09, 0.09, and 0.05 and *p* values were 0.90, 0.98, and 0.99; for *B*, *E*, *H*: 48 h, F values were 0.09, 0.10, and 0.11 and *p* values were 0.89, 0.98, and 0.99; for *B*, *E*, *H*: 72 h, F values were 0.15, 0.05, and 0.07 and *p* values were 0.96, 0.94, and 0.99; for *C*, *F*, *I*: 0 h, F values were 0.05, 0.22, and 0.02 and *p* values were 0.99, 0.79, and 0.99; for *C*, *F*, *I*: 24 h, F values were 78.10, 2894, and 66.80 and *p* values were <0.0001 in all cases; for *C*, *F*, *I*: 48 h, F values were 704,6, 2442, and 169 and *p* values were <0.0001 in all cases; for *C*, *F*, *I*: 72 h, F values were 893.70, 3707, and 240.1 and *p* values were <0.0001 in all cases) with post-hoc Tukey's test for comparison between the data corresponding to the same time of evaluation of proliferation (0, 24, 48, or 72 h) (∗*p* < 0.0001 compared with the corresponding control [cells not treated with melatonin]).

MDA-MB-231 cell proliferation has been reported to be strongly dependent on SOCE (7, 29). Hence, we have investigated the effect of melatonin on MDA-MB-231 cell proliferation. Cells were treated with increasing concentrations of melatonin (10–1000 nM) for 48, 72, and 168 h, and subsequently, cell proliferation was assessed at time = 0 h, as well as 24, 48, and 72 h later. As shown in Figure 6, *D* and *G*, treatment with melatonin for 72 and 168 h significantly attenuated MDA-MB-231 cell proliferation at almost all the concentrations investigated, as compared with cells treated with vehicle (*p* < 0.0001; n = 6). No significant effect was observed after treatment with melatonin for 48 h (Fig. 6*A*). Interestingly, expression of exogenous TRPC6 completely reversed the effect of melatonin on cell proliferation (Fig. 6, *B*, *E* and H) while expression of TRPC6dn was without effect on the inhibitory effect of melatonin on MDA-MB-231 cell proliferation (Fig. 6, *C*, *F*, and *I*). These findings indicate that the antiproliferative effect of melatonin in MDA-MB-231 cells is overcome by the expression of functional TRPC6.

### Pharmacological inhibition or silencing of TRPC6 impairs the effect of melatonin on SOCE in MDA-MB-231 cells

**Figure 7:**
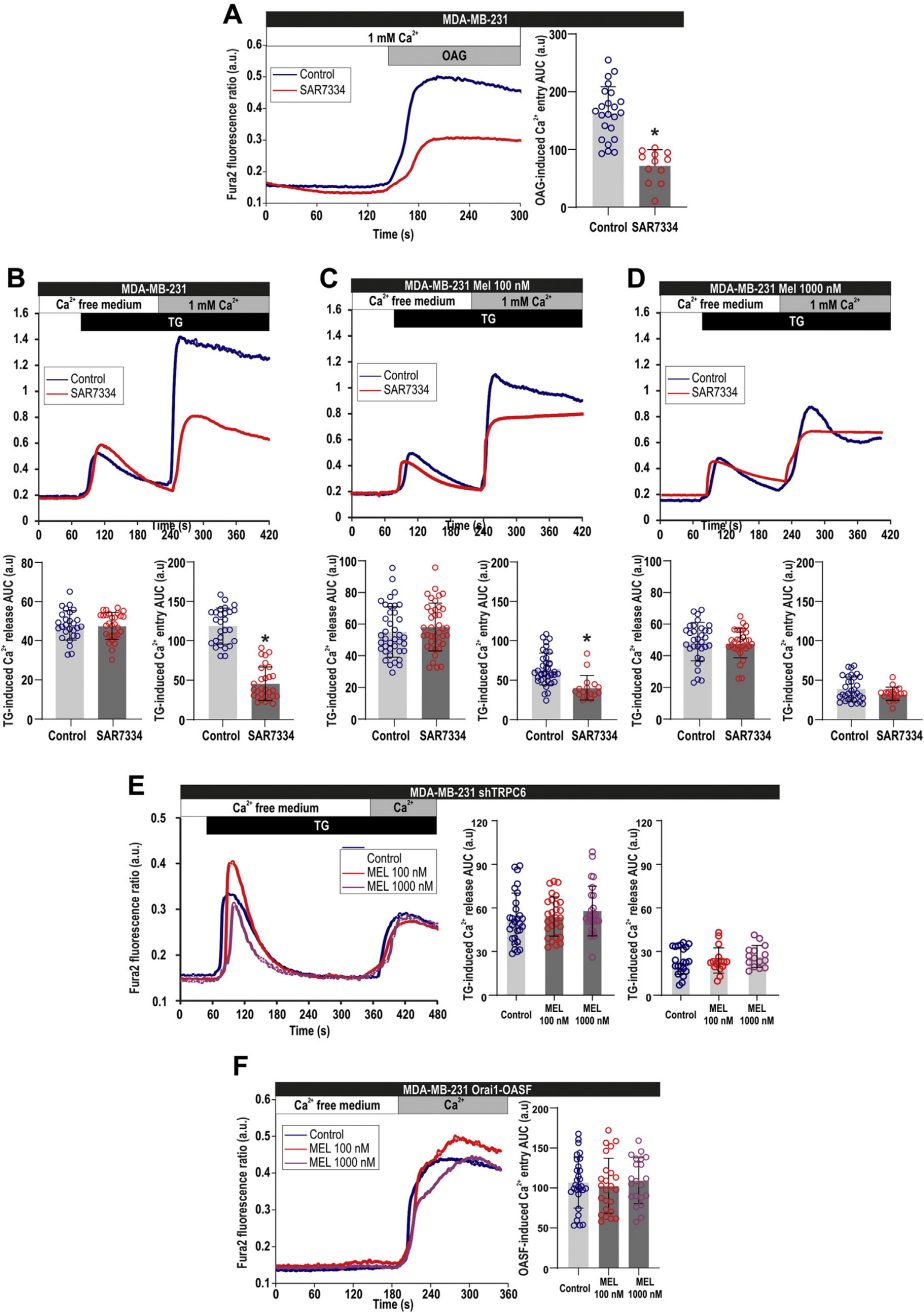
**Effect of pharmacological inhibition or silencing of TRPC6 on Ca**^**2+**^**entry in the presence of melatonin in MDA-MB-231 cells.***A*, Fura-2-loaded MDA-MB-231 cells were pretreated for 10 min with SAR7334 (1 μM). Cells were then stimulated in the presence of 1 mM extracellular Ca^2+^ with OAG (100 μM). Scatter plots represent the quantification of OAG-induced Ca^2+^ entry determined as described in Experimental procedures. Data are presented as mean ± SD. *Dots* represent a single experiment including 20–30 cells. Analysis of statistical significance was performed using Student's *t*-test (∗*p* < 0.0001 as compared with control). *B*–*D*, MDA-MB-231 cells were treated for 72 h with melatonin (100–1000 nM) or the vehicle (control). The day of the experiment, fura-2-loaded cells were pretreated for 10 min with SAR7334, were perfused with a Ca^2+^-free medium (100 μM EGTA added) and then stimulated with TG (1 μM) followed by reintroduction of external Ca^2+^ (final concentration 1 mM) to initiate Ca^2+^ entry. Data are presented as mean ± SD. *Dots* represent a single experiment including 20–30 cells. Analysis of statistical significance was performed using Student's *t*-test (∗*p* < 0.0001 as compared with control). *E*, MDA-MB-231 cells were treated with melatonin (100–1000 nM) or the vehicle (control) for 72 h. Forty-eight hours before the end of the treatment period, cells were transfected with shTRPC6. Fura-2-loaded cells were perfused with a Ca^2+^-free medium (100 μM EGTA added) and then stimulated with TG (1 μM) followed by reintroduction of external Ca^2+^ (final concentration 1 mM) to initiate Ca^2+^ entry. Scatter plots represent the quantification of TG-evoked Ca^2+^ release and entry determined as described in Experimental procedures. Data are presented as mean ± SD. *Dots* represent a single experiment including 20–30 cells. Analysis of statistical significance was performed using one-way ANOVA (F = 0.74/*p* = 0.47 and F = 0.63/*p* = 0.53 for Ca^2+^ release and entry, respectively; with post-hoc Dunnett's test for comparison between the data corresponding to Ca^2+^ entry [∗*p* < 0.0001 as compared with control]). *F*, MDA-MB-231 cells were treated with melatonin (100–1000 nM) or the vehicle (control) for 72 h. Forty-eight hours before the end of the treatment period, cells were cotransfected with Orai1 and OASF. The day of the experiment, fura-2-loaded cells were perfused with a Ca^2+^-free medium (100 μM EGTA added), followed by reintroduction of external Ca^2+^ (final concentration 1 mM) to initiate Ca^2+^ entry. Scatter plots represent the quantification of Ca^2+^ entry. Data are presented as mean ± SD. *Dots* represent a single experiment including 20–30 cells. Analysis of statistical significance was performed using one-way ANOVA (F = 0.21; *p* = 0.80).

To further support the involvement of TRPC6 in the inhibitory effect of melatonin on SOCE, we have impaired TRPC6 function either by pharmacological inhibition or by silencing the expression of the channel. As shown in Figure 7*A*, pretreatment of MDA-MB-231 cells for 10 min with the TRPC6 inhibitor SAR7334 (1 μM) significantly attenuates Ca^2+^ influx induced by 1-oleoyl-2-acetyl-sn-glycerol (OAG). In agreement with our previous studies (7), treatment of MDA-MB-231 cells with SAR7334 significantly attenuated SOCE without having any effect on TG-evoked Ca^2+^ release from the intracellular stores (Fig. 7*B*). As shown in Figure 3, treatment with 100 and 1000 nM melatonin for 72 h significantly reduced SOCE, but, interestingly, in cells pretreated with SAR7334 SOCE was not significantly different in control and upon treatment with 100 or 1000 nM melatonin (one-way ANOVA; F = 1.97 and *p* = 0.14), thus indicating that inhibition of TRPC6 channels impairs the inhibitory effect of melatonin on SOCE (Fig. 7, *B*–*D*). Similarly, we have found that cell transfection with shTRPC6, which significantly attenuates SOCE (see Fig. 7*B* *versus* Fig. 7*E*) as previously reported (7), abolished any further inhibitory effect of melatonin, as observed by treatment with 100 and 1000 nM melatonin for 72 h. Altogether, these findings support a relevant role for TRPC6 in the inhibitory effect of melatonin on SOCE in MDA-Mb-231 cells.

As previously shown in Figure 4, treatment with melatonin did not significantly alter Orai1 expression at the time and concentrations used in this study. We have further explored whether melatonin is able to alter Orai1 function. To address this issue, we have cotransfected cells with Orai1 and the Orai1-activating small fragment (OASF; amino acids 233–474) of STIM1, which is able to activate Orai1 independently on store depletion. Coexpression of Orai1 and OASF in MDA-MB-231 cells resulted in a robust activation of Ca^2+^ influx, which was unaffected by pretreatment for 72 h with melatonin 100 or 1000 nM (Fig. 7*F*). These findings indicate that melatonin does not significantly affect Orai1 function in these cells.

### Melatonin enhances the degradation of TRPC6 in MDA-MB-231 cells

**Figure 8:**
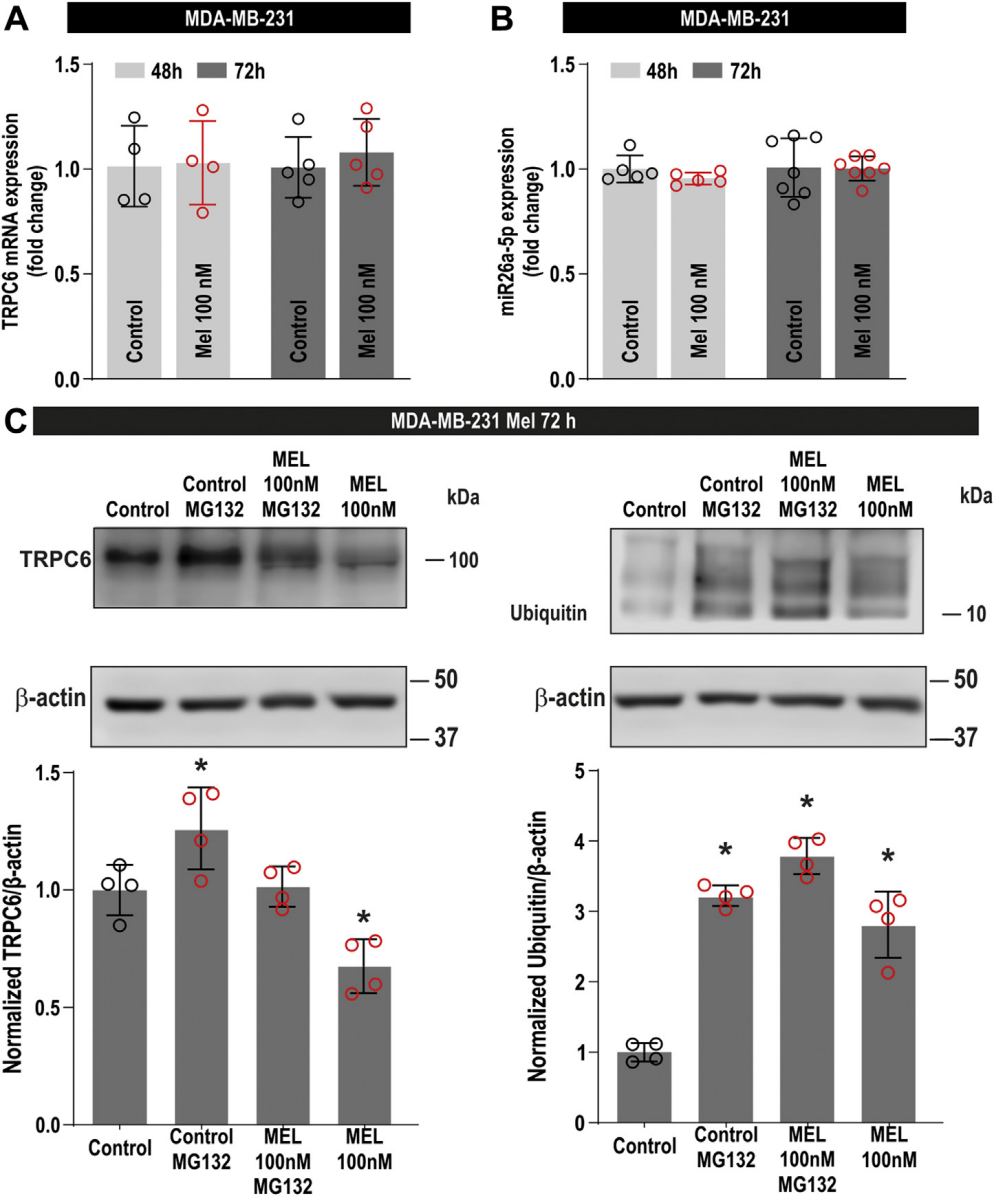
**Effect of melatonin on TRPC6 synthesis and degradation in MDA-MB-231 cells.***A* and *B*, MDA-MB-231 cells were treated for 48 or 72 h with melatonin (100 nM) or the vehicle (control). The expression of TRPC6 (*A*) or miR26a-5p (*B*) was determined as described in Experimental procedures. *Scatter plots* represent relative expression as compared with control (n = 4–7). Analysis of statistical significance was performed using one-way ANOVA (F and *p* values were 0.41 and 0.74, respectively, for [*A*] and 0.17 and 0.91, respectively, for [*B*]). *C*, MDA-MB-231 cells were treated for 72 h with melatonin (100 nM) or the vehicle (control) and lysed. Sixteen hours before the end of the treatment, cells were treated with 10 μM MG132 or the vehicle. Whole-cell lysates were immunoprecipitated with anti-TRPC6 antibody. Immunoprecipitates were subjected to 10% SDS-PAGE and subsequent western blotting with specific anti-TRPC6 (left panel), antiubiquitin (right panel), or anti-β-actin antibody, as indicated. The panels show results from one experiment representative of three others. Molecular masses indicated on the right were determined using molecular-mass markers run in the same gel. Bar graphs represent the expression of TRPC6 (left panel) and the quantification of TRPC6-associated ubiquitin. Results are presented as arbitrary optical density units and expressed as percentage of control. Analysis of statistical significance was performed using one-way ANOVA (F = 14.71 (*p* = 0.0003) and 71.28 (*p* < 0.0001) for [*C*], left and right panel, respectively) with post-hoc Dunnett's test (∗*p* < 0.05 compared with control).

We have further explored the mechanism involved in TRPC6 downregulation by melatonin. Protein expression is subjected to protein synthesis and degradation. First, we have investigated the effect of melatonin on TRPC6 synthesis by testing its possible effect on the expression of TRPC6 at the transcript level. As depicted in Figure 8*A*, qRT-PCR analysis indicates that melatonin (100 nM) was unable to alter TRPC6 mRNA expression after treatment for 48 or 72 h (n = 4–7). Hence, we have further explored the ability of melatonin to alter the expression of two miRNAs that have been reported to suppress TRPC6 expression at the protein level, miR26a-5p and miR181b (30, 31). The latter was found to be scarcely expressed in MDA-MB-231 cells, and the expression of miR26a was found to be unaffected after treatment with melatonin for 48 or 72 h (Fig. 8*B*). These findings suggest that melatonin does not alter the synthesis of TRPC6 in MDA-MB-231 cells. Therefore, we explored the effect of melatonin on TRPC6 protein degradation by analyzing TRPC6 ubiquitination. Ubiquitination was analyzed by immunoprecipitation of cell lysates with anti-TRPC6 antibody followed by western blotting with antiubiquitin antibody. Cells were treated in the absence and presence of MG132, a proteasome inhibitor that allows accumulation of the ubiquitinated form of the protein. As shown in Figure 8*C* (right panel), a small amount of ubiquitin was found to be associated to TRPC6 in untreated cells (control). Treatment with 100 nM melatonin for 72 h significantly increases the amount of ubiquitin associated to TRPC6. As expected, MG132 enhances the amount of ubiquitinated TRPC6 in cells treated with melatonin or the vehicle. Consistent with the results presented above, Figure 8*C* (left panel) reveals that TRPC6 expression was significantly attenuated in melatonin-treated cells, while addition of MG132 resulted in enhanced TRPC6 expression, due to inhibition of protein degradation, as compared with their respective controls. Altogether, these findings indicate that melatonin attenuates TRPC6 expression by enhancing protein degradation.

### Melatonin attenuates TRPC6 expression, SOCE, cell viability, and migration in the triple-negative breast cancer MDA-MB-468 cells

**Figure 9:**
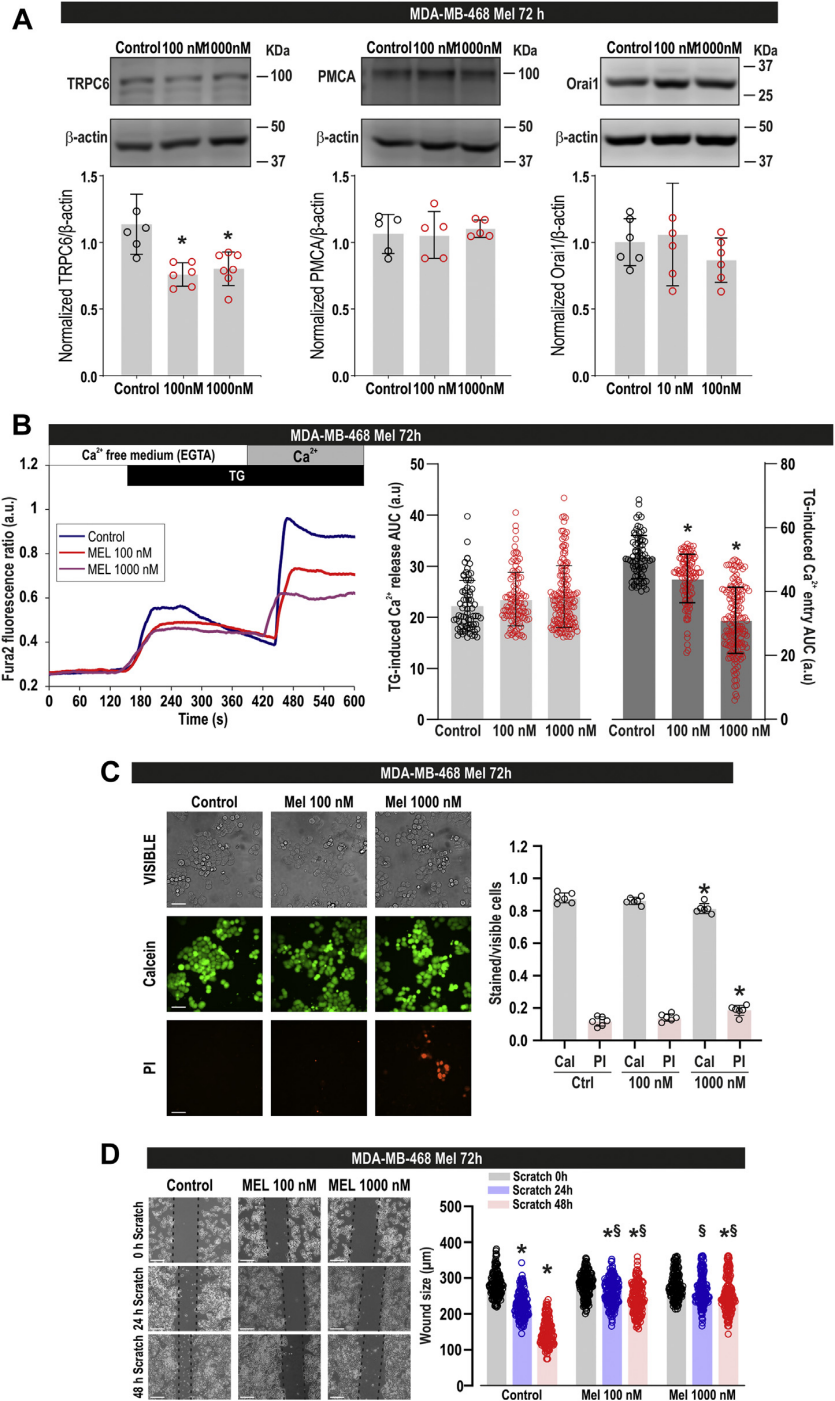
**Effect of melatonin on calcium mobilization, viability, and migration in MDA-MB-468 cells.***A*, MDA-MB-468 cells were treated for 72 h with melatonin (100–1000 nM) or the vehicle (control) and lysed. Whole-cell lysates were subjected to 10% SDS-PAGE and western blotting with anti-TRPC6, anti-PMCA, anti-Orai1, or anti-β-actin antibody, as described in Experimental procedures. Blots are representative of five to six separate experiments. Scatter plots represent TRPC6, Orai1 or PMCA expression. Analysis of statistical significance was performed using one-way ANOVA (F = 10.72, 0.16, and 0.85 and *p* = 0.001, 0.84, and 0.44 for TRPC6, PMCA, and Orai1, respectively) with post-hoc Dunnett's test for TRPC6 (∗*p* < 0.01 compared with control). *B*, MDA-MB-468 cells were treated for 72 h with melatonin (100–1000 nM) or the vehicle (control). Fura-2-loaded cells were perfused with a Ca^2+^-free medium (100 μM EGTA added) and then stimulated with TG (1 μM) followed by reintroduction of external Ca^2+^ (final concentration 1 mM) to initiate Ca^2+^ entry. Scatter plots represent the quantification of TG-evoked Ca^2+^ release and entry determined as described in Experimental procedures and are expressed as mean ± SD. *Dots* represent single experiments including 20–30 cells. Analysis of statistical significance was performed using one-way ANOVA (F values were 1.28 and 143.85, for Ca^2+^ release and entry, respectively; *p* = 0.27 and <0.0001, for Ca^2+^ release and entry, respectively,) with post-hoc Tukey test (∗*p* < 0.0001 compared with the response observed in vehicle-treated cells). *C*, MDA-MB-468 cells were treated with melatonin (100–1000 nM) or the vehicle, and 72 h later cells were loaded with calcein and propidium iodide (PI), and staining was visualized using an inverted microscope. Scatter plots represent calcein (Cal) and propidium iodide (PI) staining under the different conditions expressed as the ratio between stained and visible cells and are expressed as mean ± SD. Images shown are representative of six independent experiments. Analysis of statistical significance was performed using one-way ANOVA (for calcein data, F = 8.59 and *p* = 0.003; for PI data, F = 9.68 and *p* = 0.002) with post-hoc Dunnett's test (*p* values in the text). ∗*p* < 0.01 as compared with their respective controls (Dunnett's test). *D*, MDA-MB-468 cells were treated for 72 h with melatonin (100–1000 nM) or the vehicle (control) and were subjected to wound healing assay. Representative images were acquired at 0, 24, and 48 h from the beginning of the assay. The dotted lines define the areas lacking cells. Scatter plots represent the quantification of the wound size at the different conditions, expressed as the mean ± SD of four independent experiments. Analysis of statistical significance was performed using one-way ANOVA (F = 165.794, *p* <0.0001) with post-hoc Tukey test (∗*p* < 0.0001 compared with the scratch time = 0 h. ^$^*p* < 0.001 compared with the corresponding time in vehicle-treated cells).

We have further explored whether the effects induced by melatonin in MDA-MB-231 cells can also be extrapolated to other TNBC cells. To address this issue, we have tested the ability of melatonin to alter TRPC6 expression and different cellular functions in MDA-MB-468 cells. As depicted in Figure 9*A*, treatment of MDA-MB-468 cells for 72 h with 100 and 1000 nM melatonin significantly attenuated TRPC6 expression, with similar effect obtained by both concentrations of melatonin. By contrast, as for MDA-MB-231 cells, the expression of Orai1 and PMCA was not modified by melatonin treatment (Fig. 9*A*). Consistent with the reduction of TRPC6 expression, we have found that cell treatment for 72 h with 100 and 1000 nM melatonin significantly attenuated SOCE, without having any effect on TG-induced Ca^2+^ release from the intracellular stores (Fig. 9*B*). Furthermore, we have found that treatment for 72 h with 1000 nM melatonin significantly attenuated cell viability (Fig. 9*C*; *p* = 0.002 and 0.001 for calcein and PI staning, respectively; one-way ANOVA combined with Dunnett's test), while treatment with 100 nM melatonin had a negligible effect, if any, on the viability of MDA-MB-468 cells (Fig. 9*C*; *p* = 0.43 and 0.24 for calcein and PI staning, respectively; one-way ANOVA combined with Dunnett's test). Finally, our results indicate that melatonin at 100 or 1000 nM significantly reduces the ability of MDA-MB-468 cells to migrate (Fig. 9*D*). Therefore, our results indicate that melatonin induces similar effects in MDA-MB-231 and MDA-MB-468 cells, suggesting that these effects might be specific on TNBC cells.

## Discussion

Breast cancer is a heterogeneous disease that comprises a variety of biological subtypes with different aggressiveness and abilities to metastasize. TNBC accounts for about 10–20% of breast carcinomas and represents a therapeutic challenge mostly due to the absence of specific pharmacological targets, such as the classical estrogen and progesterone receptors and the low HER2 expression. Current therapeutic strategies are mostly based on the use of chemotherapeutics, but a number of emerging treatments have been proposed in order to potentially reduce the mortality and side effects and improve the quality of life in patients with TNBC (32).

TNBC cells express the melatonin MT1 receptor, and MT1 positivity in TNBC cells has been found to be associated with a lower stage and a smaller tumor size at time of diagnosis (33). Furthermore, occupation of MT1 receptor by oxyprenylated compounds has been demonstrated to reduce proliferation and migration of cells corresponding to a number of breast cancer subtypes, including TNBC (34). In a xenograft model of TNBC, where MDA-MB-231 cells were implanted in athymic nude mice, treatment with melatonin significantly attenuated tumor growth and angiogenesis (35). Melatonin has been reported to attenuate TNBC invasiveness by enhancing the expression of kisspeptin, a suppressor of metastasis, by activation of GATA binding protein 3 (23). Conjugation of melatonin with the estrogen receptor antagonist tamoxifen has been reported to be effective against tamoxifen-resistant MCF-7 luminal breast cancer cells and TNBC cells (36). A role for melatonin in the modulation of miRNAs in TNBC cells has been reported. Melatonin increases the gene level of miR-148a-3p and decreased the gene and protein expression of the angiogenic factors IGF-1R and VEGF in MDA-MB-231 cells, both *in vitro* and *in vivo* (37). Furthermore, melatonin upregulates antimetastatic miR-148b and the oncogenic miR-210 in MDA-MB-231 cells, although the functional significance of this response is still uncertain as depletion of the miRNAs did not abolish the effect of melatonin on migration (38).

Our results indicate that treatment with nanomolar concentrations of melatonin significantly reduces MDA-MB-231 and MDA-MB-468 cell viability, migration, and/or proliferation in a time- and concentration-dependent manner, which further supports previous studies reporting antitumoral effects of melatonin in TNBC cells (19, 34). Here we show for the first time that melatonin impairs SOCE in TNBC cells without having any significant effect on Ca^2+^ release from the intracellular stores induced by TG, which indicates that melatonin does not alter the ability of the cells to accumulate Ca^2+^ into the ER or the ER Ca^2+^ leakage rate. The abovementioned effects of melatonin were found to be specific for TNBC cells, as melatonin was without effect on nontumoral breast epithelial MCF10A cells at any of the pretreatment times and concentrations tested. Based on the previous observation that expression of functional TRPC6 plays a relevant role in the activation of SOCE in MDA-MB-231 cells (7), we have explored the possible role of TRPC6 in the inhibitory effect of melatonin in SOCE. Our results provide strong evidence for a role of TRPC6 in the antitumoral actions of melatonin. First, melatonin significantly attenuates TRPC6 expression in TNBC, without altering the expression of other plasma membrane proteins, such as the PMCA. Second, expression of exogenous TRPC6 abolishes the effect of melatonin on SOCE and cell proliferation, while overexpression of a pore-dead TRPC6 mutant (TRPC6dn) did not alter the effect of melatonin on cell proliferation. The use of TRPC6dn suggests that the antitumoral effect of melatonin involves ion influx through the channel. Third, pharmacological inhibition of TRPC6 or silencing by using RNAi impairs further inhibitory effects of melatonin. Previous studies have reported that melatonin and selenium reduce TRPM2 and TRPV1 channel activation through the modulation of the redox state (39), but, to our knowledge, this is the first description of the modulation of TRPC channels expression and/or function by melatonin. Finally, we have found that melatonin does not alter the expression of TRPC6 at the transcript level, which is in agreement with the lack of effect of melatonin on the expression of two miRNAs that have been previously shown to regulate TRPC6 expression, miR26a and miR181b (30, 31). On the other hand, our results indicate that melatonin enhances TRPC6 ubiquitination, which strongly suggests that melatonin downregulates TRPC6 expression by increasing its degradation instead of attenuating TRPC6 synthesis. Our results further show that treatment with melatonin at the preincubation time and concentrations used in this study does not alter Orai1 expression and function, assessed by the ability to mediate Ca^2+^ influx in the presence of the OASF region of STIM1.

Summarizing, although the contribution of other SOCE components cannot be excluded, our results provide evidence for the involvement of TRPC6 in the antitumoral mechanism of melatonin in TNBC cells.

## Experimental procedures

### Materials

Thapsigargin (TG), melatonin, rabbit polyclonal anti-β-actin antibody (catalog number A2066, epitope: amino acids 365–375 of human β-actin), bovine serum albumin (BSA), hydrocortisone (catalog number: H0888), insulin (catalog number: I9278), epidermal growth factor (catalog number: E9644), OAG and cholera toxin (catalog number: C8052) were from Sigma. Fura-2 acetoxymethyl ester (fura-2/AM) was from Molecular Probes. Rabbit polyclonal anti-TRPC6 antibody (catalog number: ACC-120, epitope corresponding to amino acid residues 573–586) was from Alomone. Rabbit polyclonal antiubiquitin antibody (catalog number: ab19247) was from Abcam. DharmaFECT kb transfection reagent was from Cultek. Mouse monoclonal anti-PMCA antibody (Clone 5F10, epitope: amino acids 724–783 of human PMCA) and Live/Dead viability/cytotoxicity kit were from Thermo Fisher. Horseradish-peroxidase-conjugated anti-mouse IgG antibody and anti-rabbit IgG antibody were from Jackson ImmunoResearch Europe Ltd. RNA control vector was from Origene. Enhanced chemiluminescence detection reagents were from Pierce. Bromodeoxyuridine (BrdU) cell proliferation assay kit was from BioVision. SAR7334 was from Quimigen. hTRPC6-YFP was a gift from Craig Montell (Addgene plasmid#21084; http://n2t.net/addgene:21084; RRID:Addgene_21084). All other reagents were of analytical grade.

### Cell culture and transfection

The MCF10A and the MDA-MB-231 cell lines were from ATCC. MCF10A cells were cultured at 37 °C with a 5% CO_2_ in Dulbecco's Modified Eagle Medium-F12, supplemented with 5% (v/v) horse serum, 0.5 μg/ml hydrocortisone, 10 μg/ml insulin, 20 ng/ml epidermal growth factor, and 100 ng/ml cholera toxin. MDA-MB-231 cells were cultured in Dulbecco's Modified Eagle Medium supplemented with 10% (v/v) fetal bovine serum. Both culture media contained 100 U/ml penicillin and streptomycin. Cells were transfected with either expression plasmids for TRPC6, the dominant-negative mutant of TRPC6 (TRPC6dn; provided by Dr Kristina Friedland, Friedrich Alexander University, Germany), or scramble plasmids, as described previously (7). All experiments performed have been approved by the Local Ethical Committee.

### Determination of cytosolic free-calcium concentration

Coverslips with fura-2-loaded cultured cells were mounted on a perfusion chamber and placed on the stage of an epifluorescence inverted microscope (Nikon Eclipse Ti2) with analysis system for videomicroscopy (NIS-Elements Imaging Software, Nikon). Cells, perfused with HEPES-buffered saline (HBS) containing (in mM): 125 NaCl, 5 KCl, 1 MgCl_2_, 5 glucose, 25 HEPES, and pH 7.4, supplemented with 0.1% (w/v) BSA, were alternatively excited at 340/380 nm, and fluorescence emission was detected at 510 nm using a cooled digital sCMOS camera (Zyla 4.2, Andor). Fluorescence ratio (F340/F380) was calculated pixel by pixel. TG-evoked Ca^2+^ release and entry were estimated as the integral of the rise in fura-2 fluorescence ratio for 2½ min after the addition of TG or CaCl_2_, respectively.

### Immunoprecipitation and western blotting

Immunoprecipitation and western blotting were performed as described previously (40). Aliquots of MDA-MB-231 lysates were immunoprecipitated by incubation with 2 μg of anti-TRPC6 antibody and 25 μl of protein A-agarose overnight at 4 °C on a rocking platform. Both immunoprecipitated and lysated samples were resolved by 10% SDS-PAGE, and proteins were transferred onto nitrocellulose membranes for subsequent probing. Residual protein binding sites were blocked by incubation for 60 min at room temperature with 10% (w/v) BSA in tris-buffered saline (in mM): 20 Tris base, 137 NaCl, pH 7.6 at 37 °C, with 0.1% Tween 20 (TBST). Immunodetection of TRPC6, PMCA, ubiquitin, and β-actin was achieved by incubation overnight with anti-TRPC6 antibody diluted 1:500 in TBST (10% BSA), for 2 h with anti-PMCA antibody diluted 1:1000 in TBST (10% BSA), overnight with antiubiquitin antibody diluted 1:1000 in TBST (10% BSA), or for 1 h with anti-β-actin antibody diluted 1:2000 in TBST (10% BSA). The primary antibody was removed and blots were washed with TBST. Blots were then incubated for 1 h with horseradish-peroxidase-conjugated goat anti-rabbit or goat anti-mouse IgG antibody diluted 1:10,000 in TBST (1% BSA) and then exposed to enhanced chemiluminiscence reagents for 5 min. The density of bands was measured using a C-DiGit Chemiluminescent Scanner (LI-COR Biosciences).

### Wound healing assay

Cells were seeded in 35-mm 6-well multidish to obtain confluence. Cells were then cultured in the presence of 1% serum, and a wound was manually made using a sterile pipette tip. Photographs were taken at the times indicated using an EVOS FL auto 2 cell imaging system (ThermoFisher Scientific). Cell migration was quantitated using Fiji ImageJ (NIH).

### Determination of cell proliferation

MCF10A and MDA-MB-231 cells were seeded into 96-well plates at a concentration of 2 × 10^3^ cells/well or 5 × 10^3^ cells/well, respectively. After 0, 24, 48, and 72 h, cell proliferation was assessed by a cell proliferation assay kit (BioVision) based on BrdU incorporation during DNA synthesis. Absorbance was measured using a plate reader (Epoch, Biotek) at 450 nm and expressed as arbitrary units.

### Determination of cell viability

Cell viability was tested using the Live/Dead viability/cytotoxicity kit as described previously (28). Briefly, cells were incubated with calcein-AM and propidium iodide (PI) following the manufacturer's instructions, and samples were excited at 430 nm and 555 nm for calcein and propidium iodide, respectively. Fluorescence emission at 542 nm (for viable cells) and 624 nm (for dead cells) was recorded using an EVOS FL auto 2 cell imaging system (ThermoFisher Scientific). Cell viability was quantitated using Fiji ImageJ (NIH).

### Total RNA extraction, reverse transcription, and real-time PCR

Total RNA was extracted from MDA-MB-231 with Quiazol reagent (QIAGEN). We used the miRNeasy kit to extract small RNAs and mRNA following the manufacturer's instructions, and miRNAs were quantified using a fluorometer Qubit 4 (Thermo Fisher Scientific Inc). For miRNA RT-qPCR, cDNA from total RNA was generated with the miScript II RT Kit (QIAGEN).

For mRNA RT-qPCR, cDNA was synthesized with 1 μg of total RNA using iScriptTM cDNA Synthesis Kit (Bio-Rad) according to the manufacturer's instructions in a total volume of 20 μl. Transcript of cDNA was analyzed in triplicate by RT-qPCR performed with Applied Biosystems ViiA 7 7900HT thermocycler using iTaq Universal SYBR Green Supermix (Bio-Rad). Relative quantification analyses were performed using the software SDS2.2 and Expression Suite Software V1.0.3 (Applied Biosystems, Thermo Fisher Scientific Inc), online software ThermoFisher Cloud and QuantStudio RT-qPCR Software (Thermo Fisher Scientific Inc), and Excel.

Fold changes of miRNA and gene expression were calculated relatively to housekeeping expression, 18S for gene and Snord95 for miRNA, using the comparative cycle threshold CT (ΔΔCT) method.

### Statistical analysis

Data were assessed for normality using the D'Agostino and Pearson test and the Shapiro–Wilk test for normality. Analysis of statistical significance was performed using one- or two-way ANOVA with either post-hoc Dunnett's or Tukey's test as appropiate. *p* < 0.05 was considered significant for a difference. GraphPad Prism 8 software was used for the statistical analyses.

## Data availability

All data are included in this article. The data sets generated and analyzed during the current study are available from the corresponding author on reasonable request.

## Conflict of interest

The authors declare that they have no conflicts of interest with the contents of this article.
